# Phase correlation imaging of unlabeled cell dynamics

**DOI:** 10.1038/srep32702

**Published:** 2016-09-12

**Authors:** Lihong Ma, Gannavarpu Rajshekhar, Ru Wang, Basanta Bhaduri, Shamira Sridharan, Mustafa Mir, Arindam Chakraborty, Rajashekar Iyer, Supriya Prasanth, Larry Millet, Martha U. Gillette, Gabriel Popescu

**Affiliations:** 1 Quantitative Light Imaging Laboratory, Department of Electrical and Computer Engineering, Beckman Institute for Advanced Science and Technology, University of Illinois at Urbana-Champaign, Urbana, Illinois 61801, USA; 2 Institute of Information Optics, Zhejiang Normal University, Jinhua, Zhejiang, 321004, China; 3 Department of Cell and Developmental Biology, University of Illinois at Urbana-Champaign, Urbana, Illinois 61801, USA; 4 Biological and Nanoscale Systems Group, Biosciences Division, Oak Ridge National Laboratory, Oak Ridge, TN, 37831, USA; 5 Neuroscience Program, Department of Cell and Developmental Biology, University of Illinois at Urbana-Champaign IL 61801, USA

## Abstract

We present phase correlation imaging (PCI) as a novel approach to study cell dynamics in a spatially-resolved manner. PCI relies on quantitative phase imaging time-lapse data and, as such, functions in label-free mode, without the limitations associated with exogenous markers. The correlation time map outputted in PCI informs on the dynamics of the intracellular mass transport. Specifically, we show that PCI can extract quantitatively the diffusion coefficient map associated with live cells, as well as standard Brownian particles. Due to its high sensitivity to mass transport, PCI can be applied to studying the integrity of actin polymerization dynamics. Our results indicate that the cyto-D treatment blocking the actin polymerization has a dominant effect at the large spatial scales, in the region surrounding the cell. We found that PCI can distinguish between senescent and quiescent cells, which is extremely difficult without using specific markers currently. We anticipate that PCI will be used alongside established, fluorescence-based techniques to enable valuable new studies of cell function.

Cells exhibit complex dynamic behavior across broad spatial and temporal scales^1^. In recent years, it has become increasingly clear that studying the cytoskeleton and its dynamic properties is central to understanding the physics of living cells throughout the cell cycle^2^. Actin, microtubules, and intermediate filaments are polymers that not only offer mechanical support to cells, but also act as tracks along which intracellular transport takes place^3^. Trafficking of vesicles and organelles along cytoskeletal structures inside cells is expected to be a combination of both diffusive and molecular-motor-driven processes^4^,^5^. In order to study the transport of discrete objects in the cell, e.g. vesicles, *particle tracking* has become a routine method^6^,^7^,^8^. However, the cell contains many extended objects or continuous media, such as actin filaments and microtubules, which, when viewed on scales larger than their mesh size, cannot be decomposed into discrete traceable objects. Thus, the spatiotemporal fluctuations of such continuous media cannot be investigated by particle tracking. To address this limitation, we have recently developed dispersion-relation phase spectroscopy (DPS)^5^,^9^,^10^ and dispersion-relation fluorescent spectroscopy (DFS)^4^,^11^, in which the continuous distribution of dry mass density or fluorophore density, respectively, is studied with a continuous model, in the frequency domain.

Currently the diffusion of fluorescently-tagged molecules is typically measured by fluorescence correlation spectroscopy (FCS)^12^,^13^,^14^,^15^,^16^,^17^ or fluorescence recovery after photobleaching (FRAP)^18^,^19^,^20^,^21^, in which the spatial scale is fixed by the excitation beam size. Image correlation spectroscopy (ICS)^22^, spatiotemporal image correlation spectroscopy (STICS)^23^, and raster image correlation spectroscopy (RICS)^24^ have been also successfully developed to infer information about fluorophore transport. STICS is complementary to ICS as it allows measuring velocity rather than just magnitude. RICS extends ICS to faster diffusion temporal scales. While very powerful, these methods are based on fluorescence imaging and, thus, are subject to phototoxicity and photobleaching limitations, which place a practical limitation on long time-scale studies. An ideal method for understanding spatiotemporal fluctuations in the living cell would cover broad scales, ~1–10^5^ nm spatially and ~1–10^5^ s temporally, which points to the need for label-free methods. In the past decade, quantitative phase imaging (QPI) has emerged as a promising approach to study cell structure and dynamics in a label-free manner^25^. Because it combines microscopy, interferometry, and holography, without exogenous contrast agents, QPI can be used to study cells over arbitrary time scales, from milliseconds to weeks^26^,^27^,^28^,^29^,^30^,^31^,^32^,^33^,^34^,^35^.

In this article, we present *phase correlation imaging (PCI)* as a label-free method based on QPI aimed at studying cell dynamics in a spatially-resolved manner. PCI outputs quantitative maps of the correlation time associated with fluctuations in the cell’s refractive index. We show that this information can reveal the diffusion coefficients of Brownian particles, without the need for particle tracking. The PCI investigation of cellular dynamics offers a detailed view of various compartments of the cell, such as in the nucleus, characterized by different time constants. PCI is extremely sensitive to mass density fluctuations at the femtogram scale^26^, which in turn report on the local dynamic properties of the cellular material. Here we show that PCI can quantify the change in actin dynamics when its polymerization is blocked by drugs and reveal that actin dynamics are subdominant at small spatial scales. Furthermore, we find that the distribution of correlation times is qualitatively different for quiescent and senescent cells, allowing us to classify these cell types with a label-free approach.

## Results

**Figure 1:**
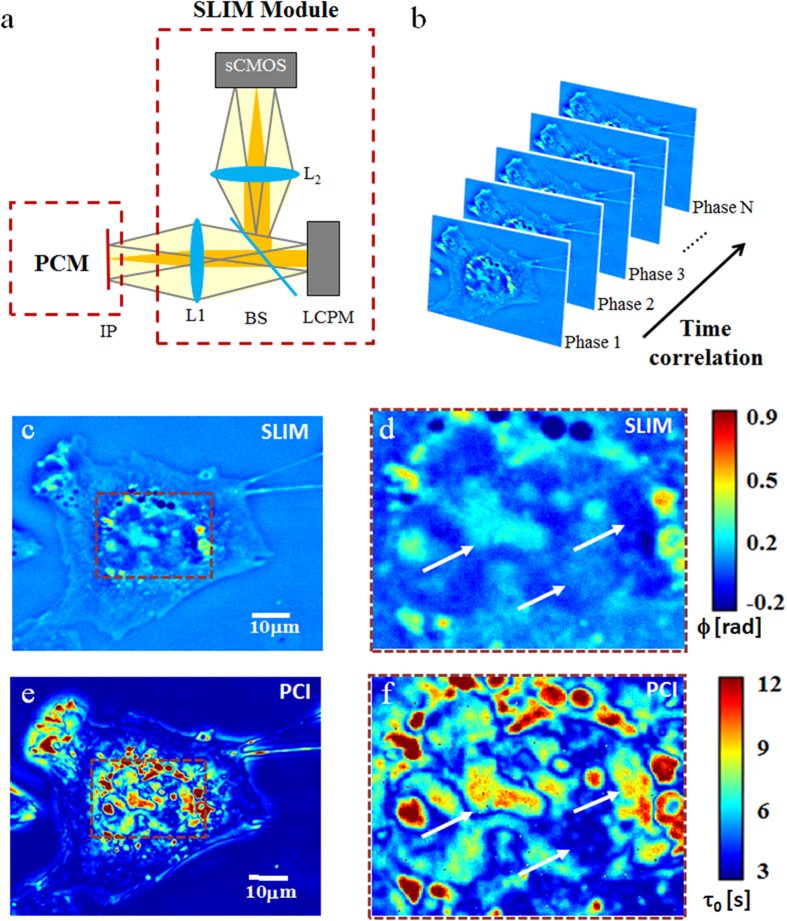
Generation of PCI maps. (**a**) SLIM module, attached to a phase contrast microscope (PCM): L1, L2, lenses, LCPM, liquid crystal phase modulator, sCMOS, complementary metal–oxide–semiconductor camera. (**b**) Schematic for generation of phase correlation image by calculating the correlation time at each pixel for a sequence of time-resolved phase images. (**c**) SLIM image of an A549 lung cancer cells. (**d**) Zoomed-in view of the cellular highlighted in c. (**e**) PCI map for the A549 ling cell in c. (**f**) PCI map corresponding to the intracellular region highlighted in e.

For imaging unlabeled live cells, we employed Spatial Light Interference Microscopy (SLIM)^36^,^37^,^38^,^39^, which is a QPI method based on phase contrast microscopy and white light illumination. Due to its broadband illumination, SLIM provides optical pathlength measurements with sub-nanometer sensitivity both spatially and temporally^38^. SLIM operates as an add-on module to a commercial phase contrast microscope (Fig. 1a), as described in more detail in Supplementary Information. In essence, our optical system makes the phase contrast objective ring appear tunable, such that one can control the phase shift between the scattered and unscattered field. In order to obtain a quantitative phase image, we record four intensity images corresponding to phase shifts that are π/2 apart^40^. Our SLIM system yields up to 12.5 quantitative phase images per second at 5.5 megapixels per frame. From the acquired time-lapse sequence, we calculate the correlation time at each pixel as illustrated in Fig. 1b and detailed in the Supplemental Information. The correlation time, τ_0_, is defined as the decay time of the temporal autocorrelation function associated with the phase fluctuations. The dynamics of the phase fluctuations report on the mass transport in the cell, as the phase map is linearly proportional to the dry mass density of the cell^26^,^41^. Large values of τ_0_ indicate slower processes, while short values denote fast dynamic regions in the cell. PCI carries the spirit of fluorescence lifetime imaging^42^, in the sense that it reveals information encoded in the time axis, which is not accessible from static imaging.

As illustrated in Fig. 1, the contrast in PCI and, thus, the ability to visually differentiate between various sub-cellular components, is determined by the difference in the dynamic properties of structures as well as the difference in optical path length. To demonstrate the ability of PCI to resolve fine sub-cellular structures, beyond what is visible from just phase images, we imaged A549 human adenocarcinoma alveolar basal epithelial cells and generated PCI maps (see Supplemental Information for details on the calculation). The cells were imaged in phenol red-free cell culture medium under physiological conditions (37° C and 5% CO_2_) with a 40X/0.75NA objective at an acquisition rate of 8 frames/s for up to 4 minutes (1,920 frames total). Representative quantitative phase images and the corresponding time correlation maps for A549 cells are shown in Fig. 1c–f. The correlation time maps, τ_0_(x, y), were obtained from the temporal autocorrelation function over sets of 100 phase images. Sliding the 100 frame set across the entire stack of 1,920 phase images, we obtain PCI maps that are *time-resolved* (see Supplemental movies S1 and S2). Figure 1e shows an example of a PCI map and Fig. 1f represents a more detailed image of the nuclear region selected in Fig. 1e. Clearly, the PCI maps are complementary to the phase images, as can be seen perhaps most noticeably in the nucleus. Low phase values can correspond to high τ_0_ values and vice versa, as the highest phase values are not necessarily the most stable. The complementarity between the SLIM and PCI maps can be seen on overlaid images in Supplementary Figs 1 and 2. The PCI maps show that the nucleus is much more fragmented from a dynamic point of view than can be glimpsed from the static, morphological images. The dynamics of nuclear material is a subject of intense research at the moment, especially in the context of gene expression^43^,^44^ and messenger RNA (mRNA) transport^45^,^46^. It is likely that PCI can become a valuable tool for studying such dynamics of crowded environments. Supplementary Videos 1 and 2 illustrate how PCI can provide complementary information to existing fluorescence approaches.

**Figure 2:**
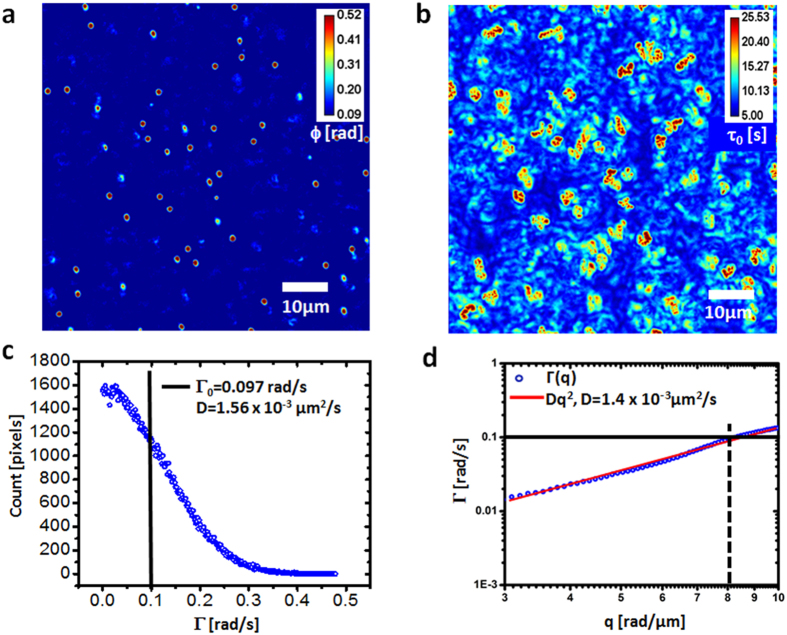
Measurement of diffusion coefficients of Brownian particles using PCI. (**a**) Quantitative phase image of 1 μm polystyrene beads in glycerol undergoing Brownian motion. (**b**) Correlation time map of the sample shown in a. (**c**) Histogram of the bandwidth values across the PCI map, band-passed around q_0_ = 8 rad/μm. The mean value is displayed using the vertical line. (**d**) Dispersion relation curve associated with the diffusive particles shown in a. The mean Γ value from c is shown by the horizontal line.

When applied to a suspension of particles under Brownian motion, we show that PCI yields the diffusion coefficient quantitatively. In order to demonstrate this capability, we imaged the Brownian motion of 1 μm polystyrene spheres in highly concentrated (99%) glycerol to mimic the viscous intracellular environment. We acquired 256 SLIM images with a 40X/0.75NA objective at an acquisition rate of 1 frame/s. Figure 2a shows a representative phase image from the time series and Fig. 2b the PCI map calculated from the entire time sequence. For Brownian motion, the dispersion relation dictates a quadratic relationship between the bandwidth, Γ, and spatial frequency, *q*, namely, Γ = D*q*^2^ (see, e.g., ref. 5). Since the information is spatially-resolved in PCI, we pick a particular spatial frequency, *q*_*0*_, around which we band-pass the PCI map, retrieve the average bandwidth from the image, Γ_0_, and obtain the diffusion coefficient as 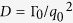. Specifically, we computed Γ from the PCI image, which was band-passed around *q*_*0*_* = 8 rad/μm,* and plotted the histogram of these bandwidth values (see Fig. 2c). The mean Γ value from the distribution is Γ_0_ = 0.097 *rad*/*s*, as indicated in Fig. 2c. As a result, the diffusion coefficient from the PCI measurement is D = (1.56 +/− 0.15) × 10^−3^ μm^2^/s. For comparison, the dispersion relation^4^,^5^, Γ *vs. q*, is shown in Fig. 2d. The quadratic fit yields a value for the diffusion coefficient, D = (1.4 +/− 0.12) × 10^−3^ μm^2^/s, which agrees well with the PCI result. Furthermore, the PCI result agrees closely with an independent particle tracking result, which yielded D = 1.6 × 10^−3^ μm^2^/s^5^.

**Figure 3:**
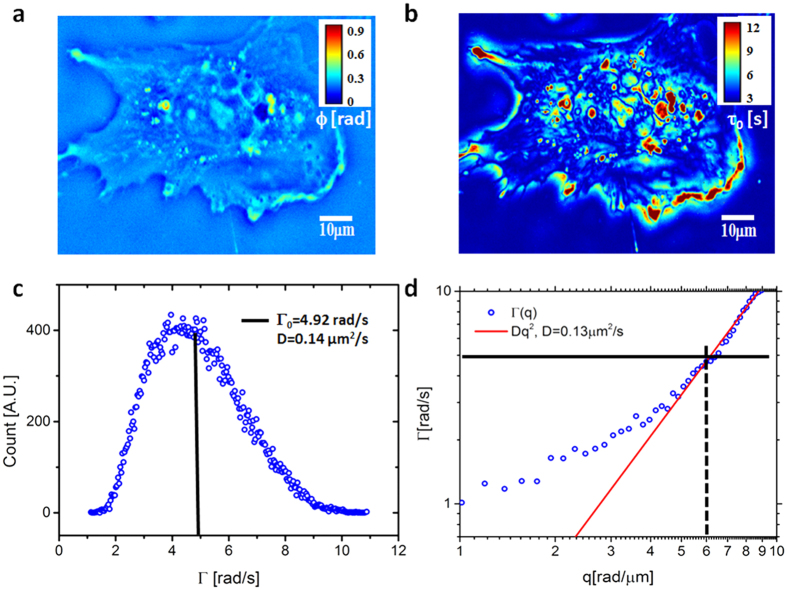
Measurement of diffusion coefficients of mass transport in live cells. (**a**) SLIM image of a A549 lung cell. (**b**) Correlation time map generated using PCI. (**c**) Histogram of the bandwidth values across the PCI map, band-passed around q_0_ = 6 rad/μm. The mean is indicated by the vertical line. (**d**) Dispersion relation curve associated with the live cell in a. The mean of Γ from c is indicated by the horizontal line.

Furthermore, we applied PCI to quantifying the dynamics of A549 lung cancer cells (Fig. 3). Band-passing the PCI map around *q*_*0*_* = 6 rad/μm* we obtained the histogram of Γ values and its mean, *Γ*_*0*_* = 4.92 rad/s,* resulted in a diffusion coefficient *D = 0.14 μm*^2^*/s* (see Fig. 3c). The dispersion relation curve shows that, indeed, at short scales (2π/*q*_*0*_*  *≤* 1 μm)*, the mass transport in the cell is diffusive. The fit with the quadratic function yielded *D = 0.13 μm*^2^*/s*, which compares very well with the PCI value. These results indicate that PCI can provide the diffusion coefficient of Brownian motion in both continuous and discrete media, without particle tracking or fluorescent markers. Our approach may become a useful complementary technique to methods such as fluorescent speckle imaging^47^.

**Figure 4:**
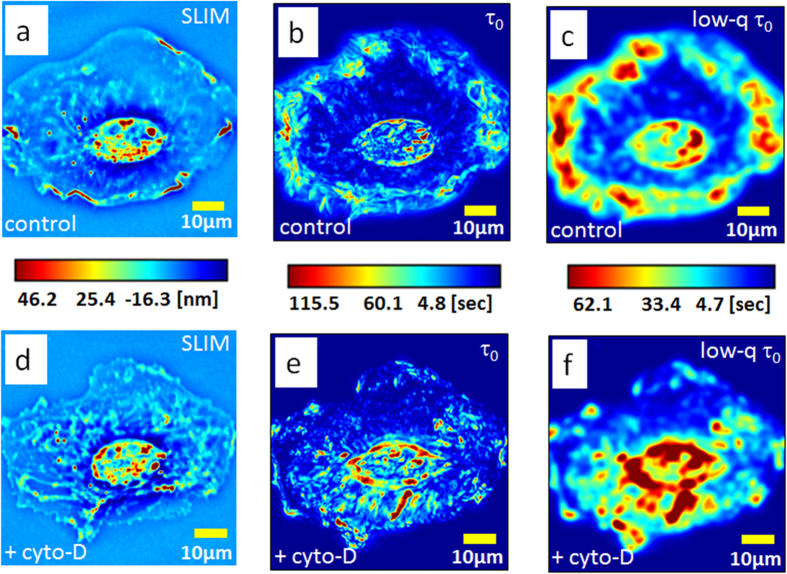
PCI studies of the effect of CytoD treatment on actin dynamics. (**a**) SLIM image of a glia cell. (**b**) The PCI map of the cell in a. (**c**) Low pass filtered version of the PCI map in b generated by blurring with a 3 μm width Gaussian kernel. (**d**) SLIM image of a glia cell after cyto-D treatment. (**e**) The PCI map of the cell in d. (**f**) Low pass filtered version of the PCI map in e generated by blurring with a 3 μm width Gaussian kernel. The color bars between each pair of figures (**a–d,b–e,c–f**) are common to the pair.

Next we demonstrate that PCI can provide unique insight on cytoskeletal dynamics (Fig. 4). We studied the lamellipodia dynamic transformations in glial cells upon treatment with cytochalasin-D (cyto-D), which is known to inhibit actin polymerization^48^. The cell culture preparation is described in more detail in Supplemental Section 5. We collected two sets of 512 SLIM images, at 1 frame/s acquisition rate, one before and another immediately after the treatment. Figure 4a–f, respectively, show the results before and after the cyto-D treatment. The ruffling lamellipodia are highlighted by PCI as regions of high τ_0_ (Fig. 4b), indicating slow dynamics. The correlation time values, of tens of seconds (Fig. 4c) are consistent with the rates of actin polymerization previously reported in the literature, e.g., 24 s in ref. 47. It is interesting to note that large τ_0_ values are exhibited at large spatial scales, as dictated by the dispersion relation. This is exhibited in Fig. 4c, in which we low-passed filtered the image in Fig. 4b by blurring (convolving) it with a Gaussian kernel of 3 μm standard deviation. In this representation, the lamellipodia dynamic is evidenced as a “crown” surrounding the cell. Interestingly, after adding cyto-D, this region of high values disappeared completely, as shown in Fig. 4f. The visual difference between the treated and un-treated cells in the correlation time map is striking, with a shift from longer times at the edges in the untreated cells to longer times in the middle in the treated cells. The actin polymerization block leaves actin predominantly in the monomeric state, evidenced here by the complete loss of the large scale ruffling at the cell periphery. The reduction in polymeric actin is characterized by much faster transport, without large scales correlations. We should clarify that, although τ_0_ is computed at short spatial scales, the τ_0_-map may contain both large and small scale features. For example, if a medium is homogeneous, the resulting τ_0_-map is constant, characterized by zero-spatial frequency, although each τ_0_-value was calculated as a high-spatial frequency quantity.

**Figure 5:**
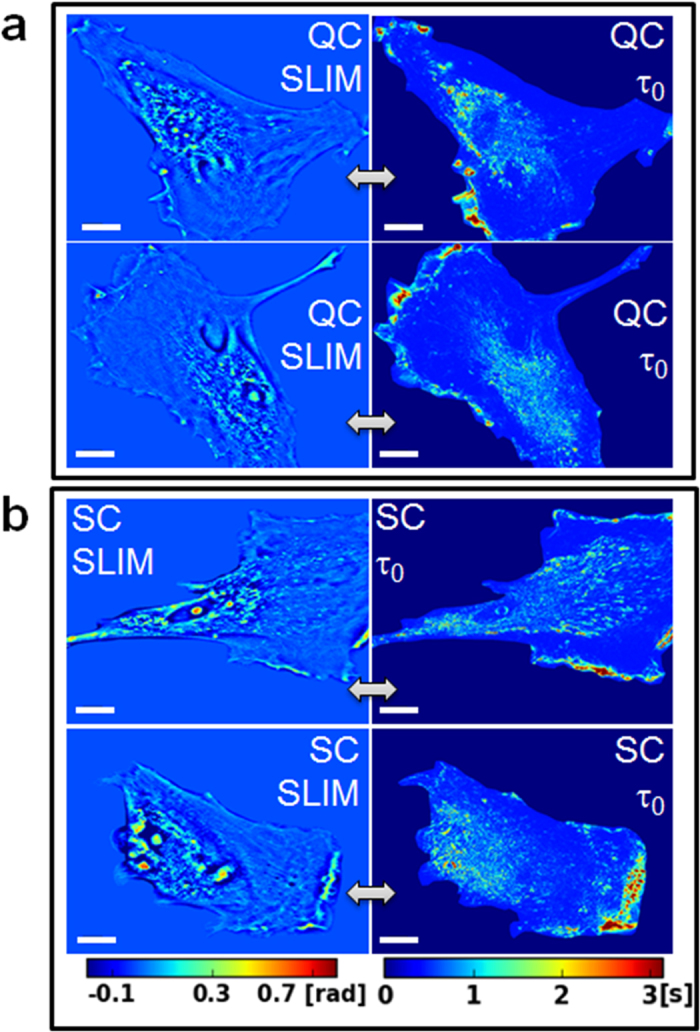
Phase correlation imaging of quiescent (QC) and senescent (SC) cells. (**a**) SLIM (left) and PCI (right) of two QC cells, as indicated. (**b**) SLIM (left) and PCI (right) of two SC cells, as indicated. The scale bar indicates 10 um. All the SLIM images and all PCI maps are on the same respective color bar, as shown.

**Figure 6:**
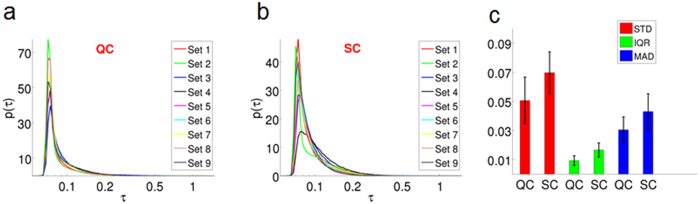
Differentiation between quiescent cells (QC) and senescent cells (SC) using PCI. (**a**) Distribution function of correlation time. p (linear axis, units of s^−1^), vs. τ_0_ (log axis, in seconds) for nine sets of QC. (**b**) Distribution function of correlation time. p (linear axis, units of s^−1^), vs. τ_0_ (log axis, in seconds) for nine sets of SC. (**c**) The median values of standard deviation (STD), inter-quartile range (IQR), and IQR and median absolute deviation (MAD), corresponding to the nine sets for QC and SC, as indicated.

We tested whether PCI is capable of distinguishing between subtle differences in the dynamics of *quiescent* and *senescent* cells. It is believed that cells in general and stem cells in particular, adopt the *quiescent* state to preserve key functional features^49^. Quiescent cells exit the cell cycle, but retain the ability to re-enter the cell cycle and undergo division. Therefore, during *quiescence,* cells (QC) can transit between a cycling and a resting state depending upon the signal they receive from the microenvironment. However, cellular *senescence* (SC) is distinct from QC, as it occurs in response to DNA damage or other cellular stress and, therefore, the cell cycle arrest is irreversible^50^. Typically senescent and quiescent cells are distinguished using the β-galactosidase assay, which requires fixation of cells^51^. Our label-free technique allows following the cells for long time periods and determining the timeline (phase of the cell cycle when the effect occurs, number of divisions before the treatment is effective) and status of cell cycle arrest (QC or SC) as a result of different treatment protocols^50^. Using SLIM, we imaged WI-38 human fibroblasts derived from lung tissue. The cells were assessed for QC and SC and compared with proliferating asynchronous cells. We imaged QC and SC cells, obtaining time-lapse stacks of 256 SLIM images, at 10 frames/s. We computed the correlation time maps for nine quiescent and nine senescent cell (see Supplemental Section 6 for details on the procedure). In Fig. 5a, we show the representative SLIM images of two different quiescent cells, and their respective correlation time maps, as indicated. Similarly, in Fig. 5b we show the SLIM images and their corresponding correlation time maps for two different senescent cells. The phase images and respective PCI maps for the additional seven quiescent and seven senescent cells are shown in Supplemental Figs S 3 and 4. To gain better insights into the nature of correlation times for the quiescent and senescent cells, we computed the probability density functions *p*(τ_0_) of the correlation time maps for each of the nine sets, which is normalized such that 
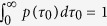
. The distributions associated with the QC and SC cells are shown in Fig. 6a,b, respectively (the τ-axis is logarithmic for simpler visualization). From these plots, it is evident that the distributions of τ_0_ (*x*, *y*) for SC cells are qualitatively broader than those of the QC cells. These findings suggest that SC cells are more likely to undergo slower intracellular trafficking than the QC counterparts. In order to quantify the spread, we computed the standard deviation (STD), inter-quartile range (IQR) and median absolute deviation (MAD) of τ_0_ (*x*, *y*) for each dataset (see Supplemental Section 6 for details). The median values over the nine sets for each group are shown in Fig. 6c. From these results, we can infer that senescent cells have greater variability in τ_0_ values, and thus higher probability of occurrence for longer correlation times. By comparison, such long-term processes are relatively rare in the quiescent cells, characterized by faster transient phenomena. These results underscore the ability of PCI for understanding the nature of temporal processes within quiescent and senescent cells and for studying cell phenotype classification.

## Summary and Discussion

We introduced a new approach for studying cell dynamics in a spatially-resolved manner. Phase correlation imaging operates on a similar principle with FLIM, in the sense that it maps spatially a parameter of dynamic relevance, the *correlation time*, τ_0_(x, y). Of course, unlike FLIM, PCI is label-free, because it exploits fluctuations in quantitative phase images due to intracellular mass traffic. Qualitatively, the correlation maps reveal dynamic subdomains in the unlabeled cells, which are independent of the morphology maps obtained from single frames. However, perhaps the most appealing feature of PCI is that it provides quantitative information from dynamic systems. For example, PCI can be used to size particles undergoing Brownian motion, essentially converting the light microscope into a dynamic light scattering instrument. Furthermore, the size of the particles can be below the resolution limit of the microscope, because PCI does not require particle tracking.

Of course, like all QPI techniques, PCI provides noninvasive investigation at the expense of specificity. However, the SLIM instrument used here has the ability to overlay the correlation maps with the fluorescence channels present in the microscope, thus providing specific ques whenever needed. Note also that the correlation time calculation is based on the inherent assumption of wide-sense stationarity, meaning that the statistical properties of the second order statistics (autocorrelation, spectrum, their moments) do not change in time. Thus, the PCI measurement must be taken over time scales that are much shorter than any scale over which the cell undergoes massive dynamic, nonstationary changes. For example, during mitosis the τ-map should be extracted from time lapse data that is much faster than, say, the one-hour interval associated with cell division.

The τ_0_ maps associated with live cells are essentially high-spatial frequency representation of the dispersion relation and, as a result, reports on the *diffusive* component of the intracellular traffic. Thus, a map of diffusion coefficients can be obtained quantitatively. This type of *diffusion-weighted imaging* of cells has the potential to underscore inhomogeneity in the mass transport at the subcellular scale. It is particularly interesting that PCI is sensitive to the integrity of actin. We measured significant changes in the τ_0_ maps due to the cyto-D block and these modifications occur predominantly at large scales. These results indicate that PCI can contribute to the field of cytoskeleton dynamics, alongside established techniques, such as fluorescence speckle imaging^44^.

Due to the nanometer sensitivity to optical pathlength changes, PCI reports on extremely subtle changes in the cell dynamics map. As an example of this capability, we showed that PCI can distinguish between that quiescent and senescent cells based on their characteristic probability density of correlation time. Such assessment is essentially impossible based on morphology alone. The common method for classifying these two types of cells relies on specific fluorescent markers. Future studies will be dedicated to analyzing dynamic variability across cell populations, for example, during the differentiation process. It will be extremely interesting to study the interplay between morphology and dynamics, structure and function, during stem cell differentiation.

It has become apparent in the past decade that quantitative phase imaging (QPI) is emerging as a valuable approach to cell biology, especially due to its label-free operation and quantitative data. The ability to combine QPI with current fluorescent techniques will be crucial for its adoption in the main stream use. Such multimodal imaging provides the benefits of both worlds: specificity associated with fluorescence, with the absence of photoxicity and photobleaching QPI. One exciting avenue is to use fluorescence as a cue for the specific structure of interest and co-localize that structure in QPI. This way, the cell dynamic behavior can be monitored over arbitrarily large temporal scales.

## Materials and Methods

### Spatial Light Interference Microscopy (SLIM)

SLIM is an add-on module that transforms an existing phase contrast microscope into a quantitative phase imaging system. As detailed in the Supplemental Information, SLIM operates by effectively turning the objective pupil ring into a tunable phase shifter, modulating this filter 4 times in increments of quarter wavelength, and collecting independent intensity images. The final quantitative phase image is obtained by combining the 4 intensity frames. SLIM preserves the diffraction resolution and all the existing channels of the microscope (e.g., fluorescence).

### Computing the correlation time maps

Using a time-lapse SLIM data set, the correlation time, *τ*_0_, is calculated at each pixel in the field of view via the variance of the temporal autocorrelation function, *τ*_0_(*x*, *y*)^2^ = <(*x*, *y*)>^2^ (see Supplemental Information for details). The bandwidth map is an entirely equivalent quantity that can be inferred from *τ*_0_ as Γ = 2*π*/*τ*_0_.

### Dispersion-relation phase spectroscopy (DPS)

The dispersion relation connects the spatial and temporal frequencies associated with the fluctuations in a SLIM time series. Thus, the temporal bandwidth for each spatial mode, *q*, has the form Γ(q) = *q*Δ*v* + D*q*^2^. The fit with the linear and quadratic term in *q*, yield the width of the velocity distribution and the diffusion coefficient, respectively (see Supplemental information for details).

### Live cell imaging

The specific procedure for each cell type is described in the Supplemental information.

## Additional Information

**How to cite this article**: Ma, L. *et al*. Phase correlation imaging of unlabeled cell dynamics. *Sci. Rep.* **6**, 32702; doi: 10.1038/srep32702 (2016).

## Supplementary Material

Supplementary Information

Supplementary Movie 1

Supplementary Movie 2

## References

[b1] Alberts B., Wilson J. H. & Hunt T. Molecular biology of the cell. 5th edn, (Garland Science, 2008).

[b2] Mofrad M. R. K. & Kamm R. D. Cytoskeletal mechanics: models and measurements. (Cambridge University Press, 2006).

[b3] Segev N. Trafficking inside cells: pathways, mechanisms, and regulation. (Landes Bioscience; Springer Science+Business Media, 2009).

[b4] Wang R., Lei L., Wang Y. X., Levine A. J. & Popescu G. Dispersion-Relation Fluorescence Spectroscopy. Physical Review Letters 109, doi: Artn 188104, doi: 10.1103/Physrevlett.109.188104 (2012). 23215337

[b5] Wang R. . Dispersion-relation phase spectroscopy of intracellular transport. Opt. Express 19, 20571–20579 (2011). 21997064 10.1364/OE.19.020571 PMC3495870

[b6] Valentine M. T. . Investigating the microenvironments of inhomogeneous soft materials with multiple particle tracking. Physical Review E 6406 (2001). 10.1103/PhysRevE.64.061506 11736190

[b7] Shin J. H., Gardel M. L., Mahadevan L., Matsudaira P. & Weitz D. A. Relating microstructure to rheology of a bundled and cross-linked F-actin network *in vitro*. P Natl Acad Sci USA 101, 9636–9641 (2004). 10.1073/pnas.0308733101 PMC470727 15210969

[b8] Waigh T. A. Microrheology of complex fluids. Rep. Prog. Phys. 68, 685–742 (2005). 10.1088/0034-4885/79/7/074601 27245584

[b9] Ceballos S. . Active intracellular transport in metastatic cells studied by spatial light interference microscopy. Journal of biomedical optics 20, 111209–111209 (2015). 26271006 10.1117/1.JBO.20.11.111209

[b10] Mir M. . Label-Free Characterization of Emerging Human Neuronal Networks. Sci. Rep. 4, doi: 10.1038/srep04434 http://www.nature.com/srep/2014/140324/srep04434/abs/srep04434.html#supplementary-information (2014). PMC3963031 24658536

[b11] Wang R. . Dispersion relations of cytoskeleton dynamics. Cell Health and Cytoskeleton 8, 1–7 (2016).

[b12] Magde D., Webb W. W. & Elson E. Thermodynamic Fluctuations in a Reacting System - Measurement by Fluorescence Correlation Spectroscopy. Physical Review Letters 29, 705-& (1972).

[b13] Digman M. A. & Gratton E. Fluorescence correlation spectroscopy and fluorescence cross-correlation spectroscopy. Wires Syst Biol Med 1, 273–282, doi: 10.1002/Wsbm.5 (2009). PMC3086279 20835996

[b14] Chen H. M., Rhoades E., Butler J. S., Loh S. N. & Webb W. W. Dynamics of equilibrium structural fluctuations of apomyoglobin measured by fluorescence correlation spectroscopy. P Natl Acad Sci USA 104, 10459–10464 (2007). 10.1073/pnas.0704073104 PMC1965535 17556539

[b15] Haupts U., Maiti S., Schwille P. & Webb W. W. Dynamics of fluorescence fluctuations in green fluorescent protein observed by fluorescence correlation spectroscopy. P Natl Acad Sci USA 95, 13573–13578 (1998). 10.1073/pnas.95.23.13573 PMC24860 9811841

[b16] Lumma D., Keller S., Vilgis T. & Radler J. O. Dynamics of large semiflexible chains probed by fluorescence correlation spectroscopy. Physical Review Letters 90, doi: 10.1103/Physrevlett.90.218301 (2003). 12786596

[b17] Bacia K., Kim S. A. & Schwille P. Fluorescence cross-correlation spectroscopy in living cells. Nature Methods 3, 83–89, doi: 10.1038/Nmeth822 (2006). 16432516

[b18] Axelrod D., Koppel D., Schlessinger J., Elson E. & Webb W. Mobility measurement by analysis of fluorescence photobleaching recovery kinetics. Biophysical Journal 16, 1055–1069 (1976). 786399 10.1016/S0006-3495(76)85755-4 PMC1334945

[b19] Yao J., Munson K. M., Webb W. W. & Lis J. T. Dynamics of heat shock factor association with native gene loci in living cells. Nature 442, 1050–1053 (2006). 16929308 10.1038/nature05025

[b20] Wang L. Y. . *In situ* measurement of solute transport in the bone lacunar-canalicular system. P Natl Acad Sci USA 102, 11911–11916 (2005). 10.1073/pnas.0505193102 PMC1187997 16087872

[b21] Politz J. C., Browne E. S., Wolf D. E. & Pederson T. Intranuclear diffusion and hybridization state of oligonucleotides measured by fluorescence correlation spectroscopy in living cells. P Natl Acad Sci USA 95, 6043–6048 (1998). 10.1073/pnas.95.11.6043 PMC27582 9600914

[b22] Petersen N. O., Hoddelius P. L., Wiseman P. W., Seger O. & Magnusson K. E. Quantitation of Membrane-Receptor Distributions by Image Correlation Spectroscopy - Concept and Application. Biophysical Journal 65, 1135–1146 (1993). 8241393 10.1016/S0006-3495(93)81173-1 PMC1225831

[b23] Hebert B., Costantino S. & Wiseman P. W. Spatiotemporal image correlation Spectroscopy (STICS) theory, verification, and application to protein velocity mapping in living CHO cells. Biophysical Journal 88, 3601–3614, doi: 10.1529/biophysj.104.054874 (2005). 15722439 PMC1305507

[b24] Mavandadi S. . Distributed Medical Image Analysis and Diagnosis through Crowd-Sourced Games: A Malaria Case Study. PLoS ONE 7, doi: 10.1371/journal.pone.0037245 (2012). PMC3350488 22606353

[b25] Popescu G. Quantitative phase imaging of cells and tissues. (McGraw-Hill, 2011).

[b26] Mir M. . Optical measurement of cycle-dependent cell growth. Proc. Nat. Acad. Sci. 108, 13124 (2011). 21788503 10.1073/pnas.1100506108 PMC3156192

[b27] Park Y. K. . Measurement of red blood cell mechanics during morphological changes. Proc. Nat. Acad. Sci. 107, 6731 (2010). 20351261 10.1073/pnas.0909533107 PMC2872375

[b28] Park Y. K. . Refractive index maps and membrane dynamics of human red blood cells parasitized by Plasmodium falciparum. Proc Natl Acad Sci USA 105, 13730 (2008). 18772382 10.1073/pnas.0806100105 PMC2529332

[b29] Kim T. . White-light diffraction tomography of unlabeled live cells. Nat Photonics 8, 256–263, doi: 10.1038/Nphoton.2013.350 (2014).

[b30] Cooper K. L. . Multiple phases of chondrocyte enlargement underlie differences in skeletal proportions. Nature 495, 375–378, doi: http://www.nature.com/nature/journal/v495/n7441/abs/nature11940.html#supplementary-information (2013). 23485973 10.1038/nature11940 PMC3606657

[b31] Park H. . Characterizations of individual mouse red blood cells parasitized by Babesia microti using 3-D holographic microscopy. Scientific reports 5, doi: 10.1038/Srep10827 (2015). PMC4650620 26039793

[b32] Cotte Y. . Marker-free phase nanoscopy. Nat Photonics 7, 113–117, doi: 10.1038/Nphoton.2012.329 (2013).

[b33] Yamauchi T., Iwai H. & Yamashita Y. Label-free imaging of intracellular motility by low-coherent quantitative phase microscopy. Opt Express 19, 5536–5550, doi: 10.1364/OE.19.005536 (2011). 21445192

[b34] Yu X. . Four-dimensional motility tracking of biological cells by digital holographic microscopy. Journal of Biomedical Optics 19, 045001–045001, doi: 10.1117/1.JBO.19.4.045001 (2014). 24699632 PMC3974550

[b35] Shaked N. T., Satterwhite L. L., Bursac N. & Wax A. Whole-cell-analysis of live cardiomyocytes using wide-field interferometric phase microscopy. Biomedical Optics Express 1, 706–719, doi: 10.1364/BOE.1.000706 (2010). 21258502 PMC3018002

[b36] Nguyen T. & Popescu G. Spatial Light Interference Microscopy (SLIM) using twisted-nematic liquid-crystal modulation. Biomedical Optics Express 4, 1571–1583 (2013). 24049678 10.1364/BOE.4.001571 PMC3771828

[b37] Bhaduri B. . Cardiomyocyte Imaging Using Real-Time Spatial Light Interference Microscopy (SLIM). Plos One 8, 0056930 (2013). 10.1371/journal.pone.0056930 PMC3574023 23457641

[b38] Wang Z. . Spatial light interference microscopy (SLIM). Opt Express 19, 1016 (2011). 21263640 10.1364/OE.19.001016 PMC3482902

[b39] Wang Z. . Spatial light interference tomography (SLIT). Opt Express 19, 19907–19918 (2011). 21996999 10.1364/OE.19.019907 PMC3495874

[b40] Wang Z. & Popescu G. Quantitative phase imaging with broadband fields. Appl Phys Lett 96, 051117 (2010).

[b41] Popescu G. . Optical imaging of cell mass and growth dynamics. Am J Physiol Cell Physiol 295, C538–C544 (2008). 18562484 10.1152/ajpcell.00121.2008 PMC2518415

[b42] Gadella T. W. J. Jr, Jovin T. M. & Clegg R. M. Fluorescence lifetime imaging microscopy (FLIM): Spatial resolution of microstructures on the nanosecond time scale. Biophysical Chemistry 48, 221–239, doi: http://dx.doi.org/10.1016/0301-4622 (93)85012-7 (1993).

[b43] Yu J., Xiao J., Ren X., Lao K. & Xie X. S. Probing gene expression in live cells, one protein molecule at a time. Science 311, 1600–1603, doi: 10.1126/science.1119623 (2006). 16543458

[b44] Hansen M. M. . Macromolecular crowding creates heterogeneous environments of gene expression in picolitre droplets. Nat Nanotechnol , doi: 10.1038/nnano.2015.243 (2015). PMC4740931 26501750

[b45] Shav-Tal Y. . Dynamics of single mRNPs in nuclei of living cells. Science 304, 1797–1800, doi: 10.1126/science.1099754 (2004). 15205532 PMC4765737

[b46] Halstead J. M. . Translation. An RNA biosensor for imaging the first round of translation from single cells to living animals. Science 347, 1367–1671, doi: 10.1126/science.aaa3380 (2015). 25792328 PMC4451088

[b47] Watanabe N. & Mitchison T. J. Single-molecule speckle analysis of Aactin filament turnover in lamellipodia. Science 295, 1083–1086 (2002). 11834838 10.1126/science.1067470

[b48] MacLean-Fletcher S. & Pollard T. D. Mechanism of action of cytochalasin B on actin. Cell 20, 329–341 (1980). 6893016 10.1016/0092-8674(80)90619-4

[b49] Sang L. Y., Coller H. A. & Roberts J. M. Control of the reversibility of cellular quiescence by the transcriptional repressor HES1. Science 321, 1095–1100, doi: 10.1126/science.1155998 (2008). 18719287 PMC2721335

[b50] Heinrichs A. Cell division - Back and forth. Nature Reviews Cancer 8, 740–740, doi: 10.1038/nrc2514 (2008).

[b51] Dimri G. P. . A biomarker that identifies senescent human cells in culture and in aging skin *in vivo*. Proceedings of the National Academy of Sciences 92, 9363–9367 (1995). 10.1073/pnas.92.20.9363 PMC40985 7568133

