# Supplementary material for: Phase correlation imaging of unlabeled cell dynamics
Source: Sci Rep. 2016 Sep 12;6:32702. doi: 10.1038/srep32702 (PMC5018886; doi:10.1038/srep32702)
Supplement: Supplementary Information [file srep32702-s1.pdf]

Supplemental Information

Phase correlation imaging of unlabeled cell dynamics

Lihong Ma[1,2], Gannavarpu Rajshekhar[1,3], Ru Wang[1], Basanta Bhaduri[1], Shamira Sridharan[1], Mustafa Mir[1], Arindam Chakraborty[4], Raj Iyer[4,6], Supriya Prasanth[4], Larry Millet[4,5], Martha U. Gillette[4,6] & Gabriel Popescu[1]

1. Quantitative Light Imaging Laboratory, Department of Electrical and Computer Engineering, Beckman Institute for Advanced Science and Technology, University of Illinois at Urbana-Champaign, Urbana, Illinois 61801, USA
2. Institute of Information Optics, Zhejiang Normal University, Jinhua, Zhejiang, 321004, China
3. Current address: Department of Electrical Engineering, Indian Institute of Technology Kanpur, Kanpur, 208016, India

4. Department of Cell and Developmental Biology, University of Illinois at Urbana-Champaign, Urbana, Illinois 61801, USA

5. Biological and Nanoscale Systems Group, Biosciences Division, Oak Ridge National Laboratory, Oak Ridge, TN, 37831, USA
6. Neuroscience Program， Department of Cell and Developmental Biology University of Illinois at Urbana-Champaign IL 61801, USA

1. Spatial Light Interference Microscopy (SLIM)

The specimens of interest were imaged with Spatial Light Interference Microscopy (SLIM) (see Ref. [1-3] for details). In short, SLIM is white-light illumination, common-path, phase-shifting interferometer, designed as an add-on module to a commercial phase contrast microscope (PCM) (Zeiss Axio Observer Z1). At the light port of PCM, SLIM module is composed of a 4f system (the focal lengths of the Lens L1 and L2 are f_1=150 mm and and f_2=150 mm). Therefore, the specimen without further magnification is imaged onto a scientific-grade complementary metal oxide semiconductor (sCMOS) camera (Andor, Zyla), which is capable of imaging at 100 frames/s, each frame of 5.5 megapixels. The back focal plane of a phase contrast objective (Zeiss, ph2, 40X, NA=0.75) is projected onto a reflective liquid crystal phase modulator (LCPM). In addition to the conventional $\boldsymbol{\pi}/2$ phase shift induced by PCM to the unscattered field, the SLIM module introduces three controllable phase shifts in increments of $\boldsymbol{\pi}/2$ by LCPM such that a unique quantitative phase image is reconstructed from the four interferograms between the scattered and unscattered fields. Note the operation protocol and the environmental control is a standard accessory for the existing commercial microscope base. By moving the objective, the specimen is precisely imaged onto the

camera plane. The difference is that the computer synchronization between the phase-shifting control and camera exposure is needed for data acquisition. In order to acquire a sequence of phase images, the acquisition process is repeated. The acquisition speed is only limited by the detector frame rate and the refresh rate of the liquid crystal phase modulation (LCPM in Fig. 1a). Our SLIM system allows fast phase imaging up to 12.5 Hz for each frame consisting of 5.5 megapixels.

2. Phase Correlation Imaging

In PCI we compute the correlation time map at each pixel from the acquired time-lapse SLIM images, which are essentially cellular dry mass density distributions. Each phase image reflects the optical path length information of living cells, which depends on both the thickness and the refractive index information. Due to intracellular transport, a combination of Brownian and deterministic motion, the phase fluctuates in time. The phase fluctuations around the average can be expressed as $\Delta\phi(\mathbf{r},t)=\phi(\mathbf{r},t)-\langle\phi(\mathbf{r},t)\rangle_t$, where $\phi(\mathbf{r}, t)$ denotes the phase distribution at time t and the angular bracket indicates temporal average. From the measured phase fluctuation data $\Delta\phi(\mathbf{r},t)$, we calculate its temporal correlation function $g(\mathbf{r},\tau)$:

$$g(\mathbf{r},\tau)=\frac{<\Delta\phi(\mathbf{r},t)\Delta\phi(\mathbf{r},t+\tau)>}{<\Delta\phi(\mathbf{r},t)>^2} \quad (1)$$

Then the correlation time $\tau_0(x, y)$ is defined as the decay time, i.e., the standard deviation of the correlation function, defined via the second order moment as,

$$\tau_0(x,y)^2=\frac{\int \tau(x,y)^2 g(r,\tau)d\tau}{\int g(r,\tau)d\tau} \quad (2)$$

Note that temporal correlation function, g, is the Fourier transform of temporal power spectrum, and the decay rate $\Gamma(x, y)$ of the power spectrum is proportional to the reciprocal of correlation time $\tau_0(x, y)$:

$$\Gamma=\frac{2\pi}{\tau_0} \quad (3)$$

3. Dispersion-relation phase spectroscopy (DPS)

Dispersion-relation phase spectroscopy (DPS) provides an ability to quantify intracellular transport in a label free manner. It has been well documented that intracellular transport includes contributions from both deterministic transport at large scales and diffusive transport at small scales. As the phase image measured by SLIM is related to the thickness and the refractive index, phase image is essentially a dry mass density map and the changes in density satisfy an advection-diffusion equation. Taking a spatial Fourier transform, the temporal autocorrelation, g, for each spatial frequency mode, q, can be expressed as:

$$g(\boldsymbol{q},\tau)=e^{iv_0 q\tau}e^{-q\Delta v\tau-\mathrm{D}q^2\tau} \quad (4)$$

Where D is diffusion coefficient, v_0 is the averaged advection speed, and Δv is the bandwidths of the speed distribution. Thus, the temporal autocorrelation decays exponentially at a rate Γ,

$$\Gamma(\mathrm{q})=q\Delta v+\mathrm{D}q^2 \quad (5)$$

The dispersion relationship between the decay rate and spatial mode can be used to estimate the mean diffusion coefficient and the bandwidth of advection speed. In practice, the decay rate Γ is calculated from the time lapse phase images.

4. Imaging A549 lung cancer cells The alveolar basal epithelial cell line, A549, was used in this study. The cells were cultured under standard growth conditions in Roswell Park Memorial Institute medium (RPMI). The cells were passaged at 70-90% confluence. For imaging, the cells were passaged to a 30-50% confluence in a glass bottom dish with colorless RPMI. The cells were imaged every 15 seconds for 5 minute intervals on the SLIM system equipped with an incubating chamber.

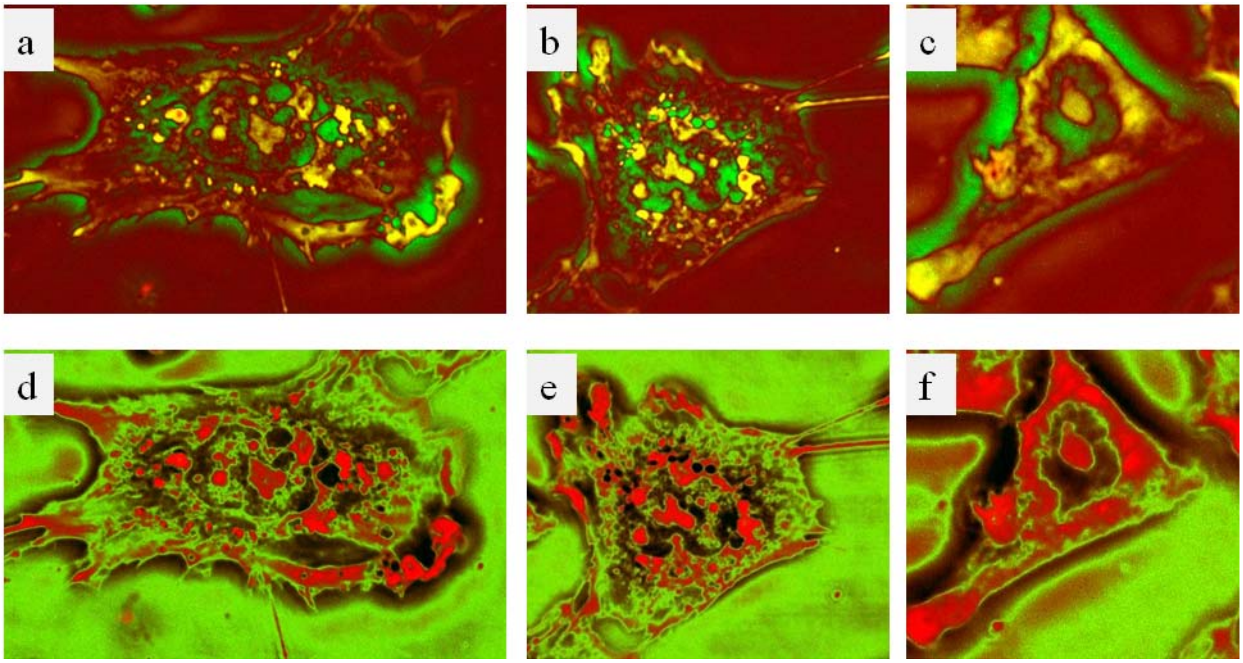

Fig. S1. *Overlay maps for three different A549 lung cancer cells. (a-c) overlay of the phase image (red channel) and the correlation time map (green channel) for three different A549 lung cancer cells, respectively. (d-f) the overlay of the phase image (red channel) and the correlation bandwidth map (green channel) for three different A549 lung cancer cells, respectively.*

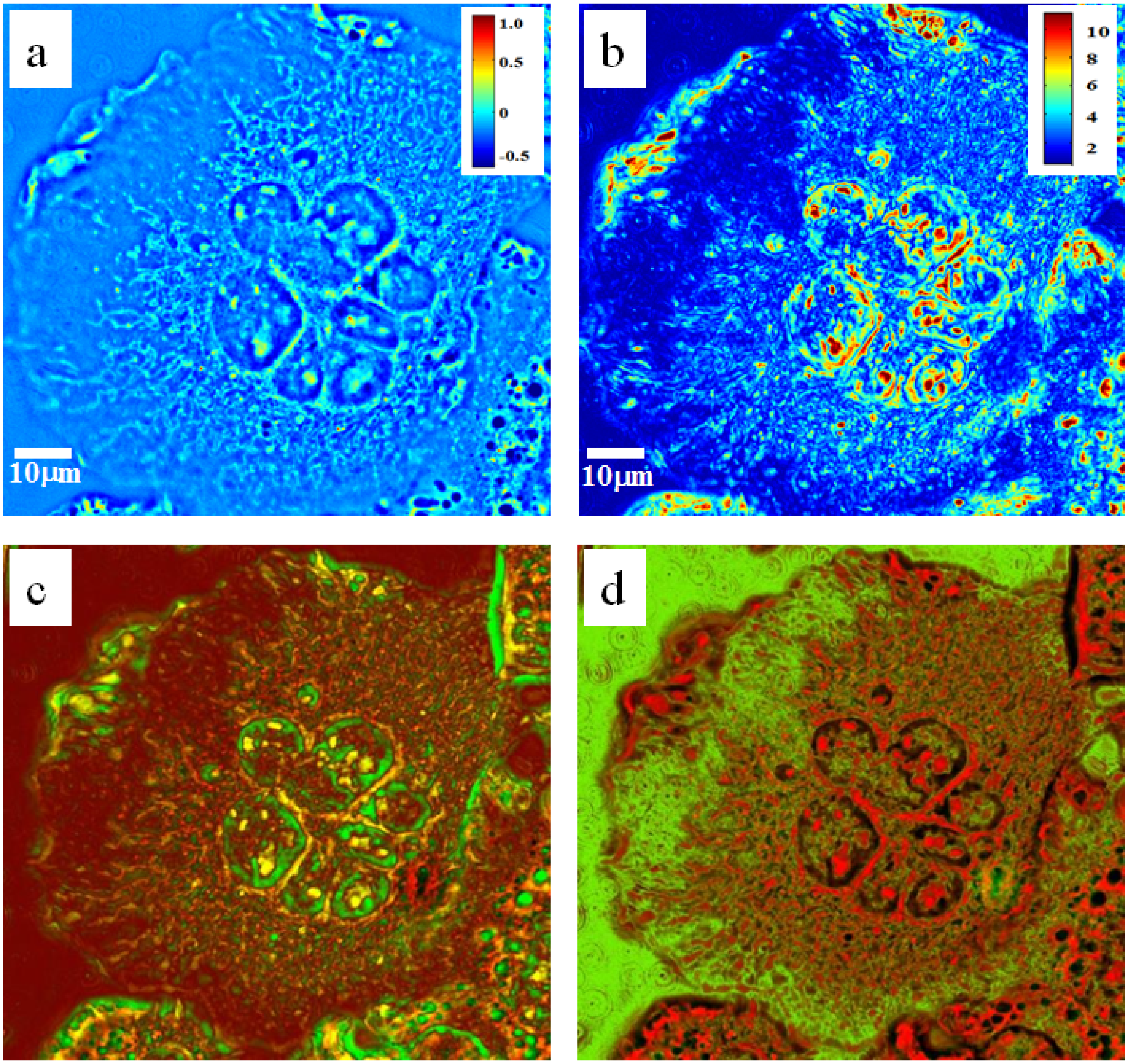

Figure S2. *(a) Phase image.(b) correlation time map. (c) overlay map of the phase image (red channel) and the correlation time map (green channel). Yellow indicates areas of overlap between the two channels. (d) the overlay map of the phase image (red channel) and the correlation bandwidth map (green channel).*

5. Imaging glia cells.

Enteric glial cells (EGC) are an integral part of the enteric (*i.e.* intestinal) nervous system. Interactions involving EGC and other cellular constituents of the enteric nervous system are implicated in complex homeostatic and inflammatory processes. Neuronal, immune, and epithelial cells, along with capillaries of the gut, coordinate with EGC to modulate motility of the gastrointestinal tract and respond to inflammation. Neuro-gliopathies may be implicated in common and debilitating gut malfunctions (e.g. Crohn's disease and inflammatory bowel disease).

Cultured EGCs (ATCC, CRL-2690, designation: EGC/PK060399egfr) were grown in DMEM (Gibco product #31053) with glucose (4.5 g/L). Growth media supplements of 10% fetal bovine serum and 3.0 mM L-glutamine were added to DMEM prior to cell culture. EGC were grown to confluence and propagated through conventional release and plating processes (release with trypsin EDTA, resuspension, dilution, then plating). For imaging studies, cells were seeded onto glass-bottom Fluorodishes (catalogue number: FD-35; World Precision Instruments, Sarasota, FL) at low densities prior to imaging. During image acquisition, EGC were maintained at physiological temperatures (37 °C) with an environmental control chamber; to maintain pH, a CO_2-independent media (Hibernate-A with 3.0mM L-glutamine and 10% fetal bovine serum) was used. Cytochalasin-D (Cyto-D, 5 μM) was used to treat the EGC cytoskeleton.

6. Imaging quiescent and senescent cells

We utilized WI38 cells to induce quiescence by serum deprivation. Briefly, WI38 cells were grown in media containing 0.1% fetal bovine serum (FBS) for 72hrs to cause a reversible cell cycle arrest or quiescence or G0 [4]. To induce senescence by replicative stress, WI38 cells were treated with doxorubicin (DOX) to a final concentration of 100ng/ml for 3 days in the media containing 10% FBS [5,6].

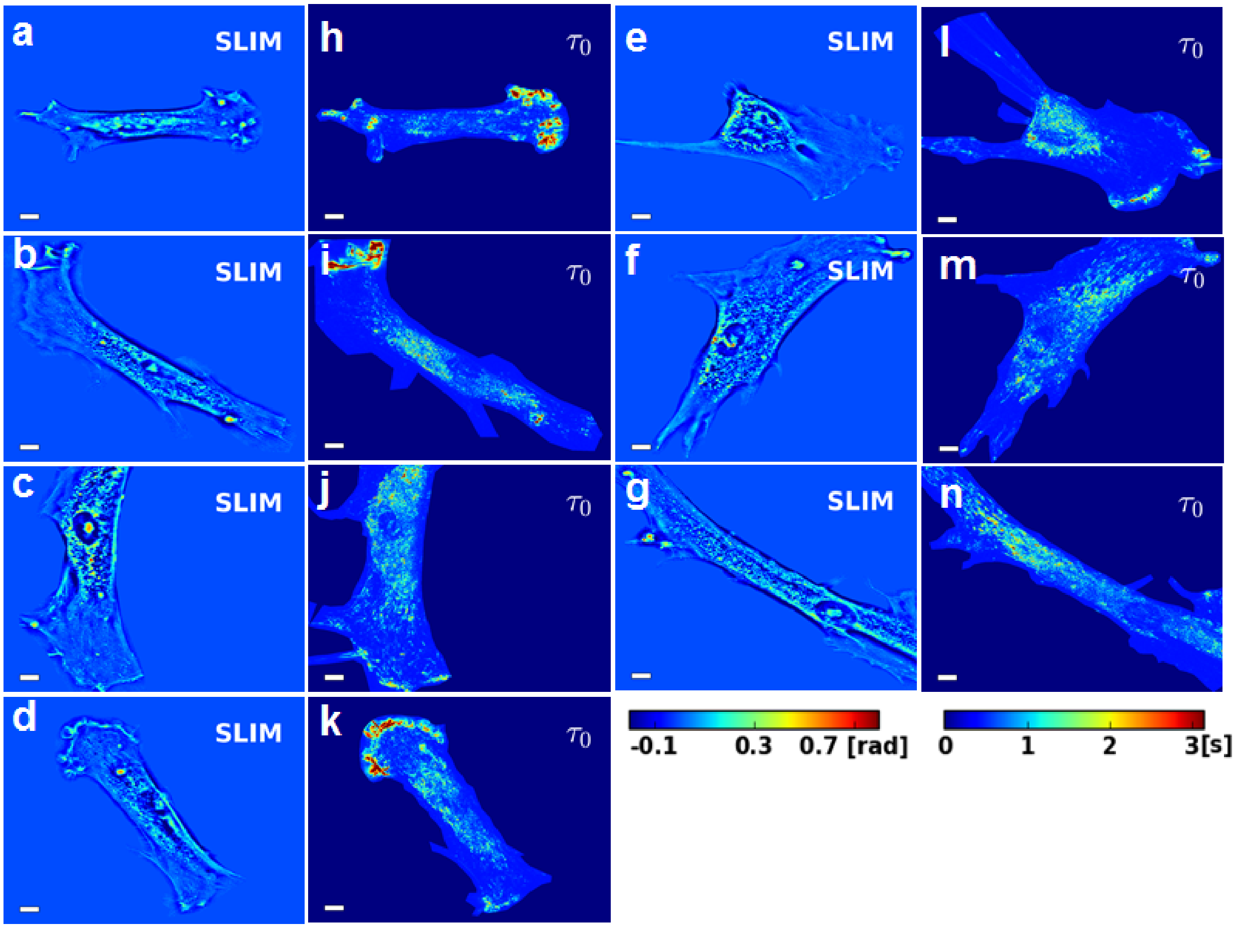

Fig. S3. *Phase correlation imaging of quiescent cells (QC). (a-g) Quantitative phase maps in radians for different quiescent cells. (h-n) The corresponding correlation time maps in seconds. The scalebar indicates 10 microns.*

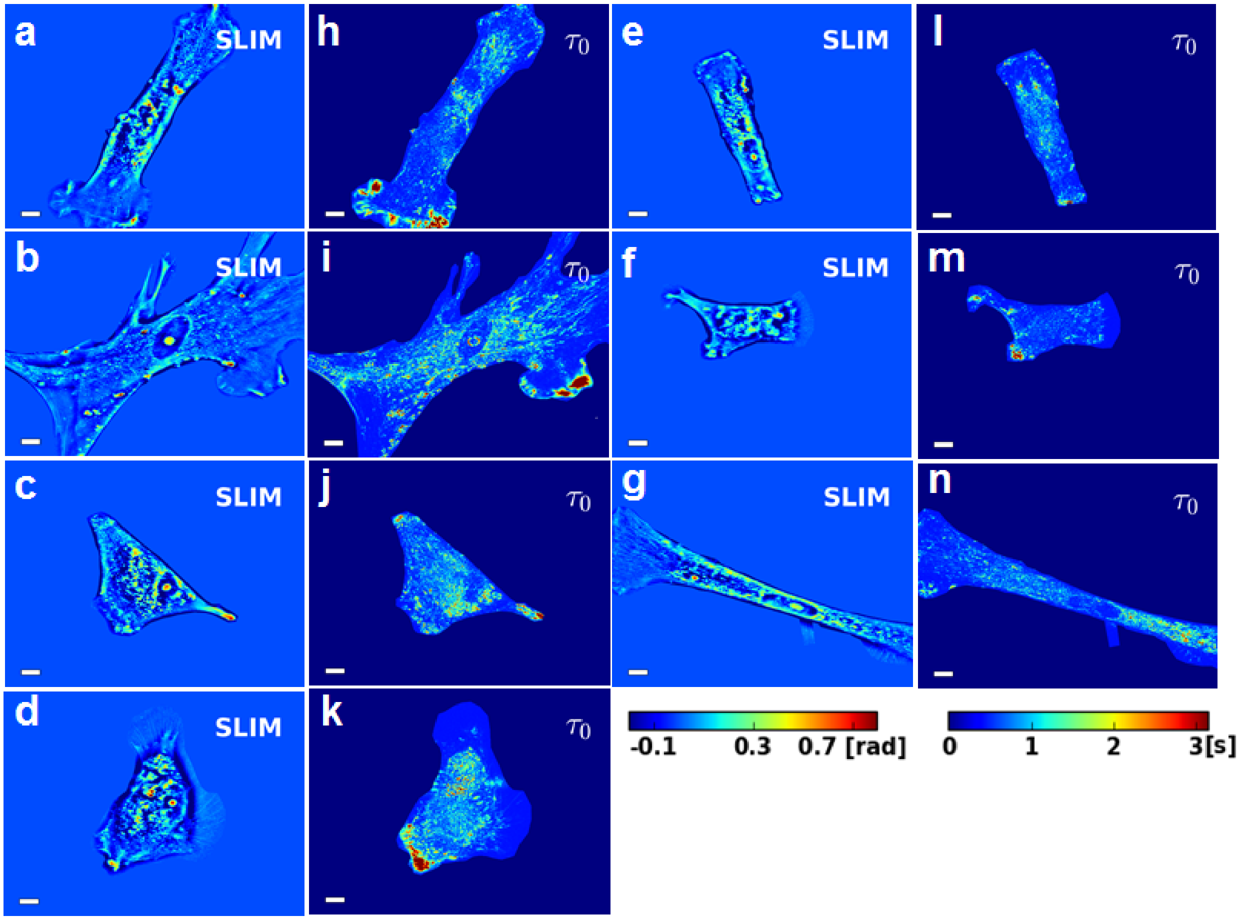

Fig. S4. *Phase correlation imaging of senescent cells (SC). (a-g) Quantitative phase maps in radians for different senescent cells. (h-n) The corresponding correlation time maps in seconds. The scalebar indicates 10 microns.*

The appearance of the "fried egg" morphology of cells indicated the induction of cellular senescence. We also utilized the β-galactosidase (β-gal) staining to confirm cellular senescence[7] [Fig. S5a]. Moreover, analysis of cellular markers like p21 and p53 either by immunoblotting [Fig. S5b] or qPCR analysis (data not shown) further indicated the doxorubicin induced cellular senescence (Demidenko and Blagosklonny, 2008) [6].

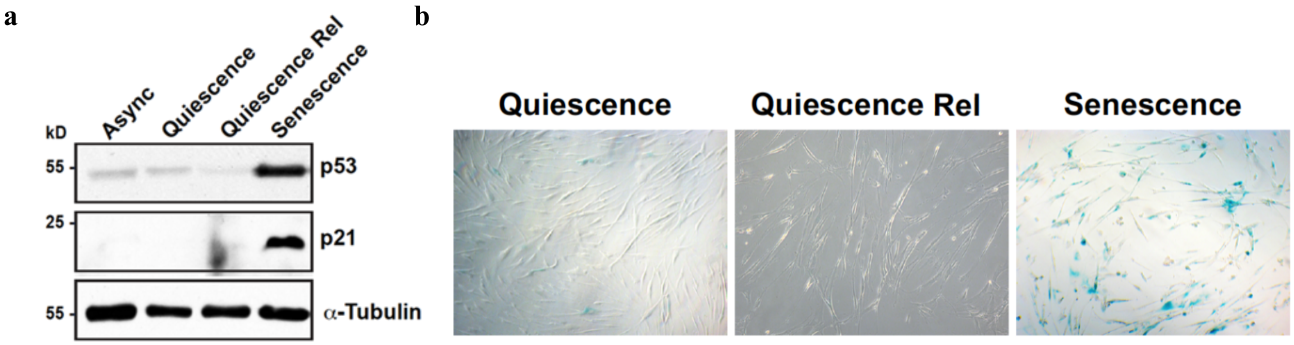

Fig. S5. *Cellular quiescence and senescence in WI38 induced by serum starvation and DOX. (a) Immunoblot analysis of WI38 cells after serum starvation (quiescence), release (quiescence release) and DOX treatment (senescence). Elevated levels of p53 and p21 have been used as the marker of senescent cells. (b) β-gal staining in the cytoplasm of senescent cells.*

We performed all image processing operations and numerical computations in MATLAB. The histogram, standard deviation (STD), median absolute deviation (MAD) and inter-quartile range (IQR) calculations were done using MATLAB's in-built functions. The histogram normalization was performed to ensure unit area probability density function.

Video Caption

Supplemental Movie 1. Overlay of phase image (red channel) and the correlation time map (green channel) for a A549 lung cancer cell.

Supplemental Movie 1. Overlay of phase image (red channel) and the correlation time map (green channel) for a A549 lung cancer cell.

REFERENCES

1 Kim, T. *et al.* White-light diffraction tomography of unlabeled live cells. *Nat Photonics* **8**, 256-263, doi:Doi 10.1038/Nphoton.2013.350 (2014).

2 Wang, Z. *et al.* Spatial light interference microscopy (SLIM). *Opt Express* **19**, 1016 (2011).

3 Wang, Z. *et al.* Spatial light interference tomography (SLIT). *Opt Express* **19**, 19907-19918 (2011).

4 Chang, H. Y. *et al.* Gene expression signature of fibroblast serum response predicts human cancer progression: similarities between tumors and wounds. *PLoS Biol* **2**, E7, doi:10.1371/journal.pbio.0020007 (2004).

5 Chang, B. D. *et al.* A senescence-like phenotype distinguishes tumor cells that undergo terminal proliferation arrest after exposure to anticancer agents. *Cancer Res* **59**, 3761-3767 (1999).

6 Demidenko, Z. N. & Blagosklonny, M. V. Growth stimulation leads to cellular senescence when the cell cycle is blocked. *Cell Cycle* **7**, 3355-3361, doi:10.4161/cc.7.21.6919 (2008).

7 Dimri, G. P. *et al.* A Biomarker That Identifies Senescent Human-Cells in Culture and in Aging Skin in-Vivo. *P Natl Acad Sci USA* **92**, 9363-9367, doi:DOI 10.1073/pnas.92.20.9363 (1995).
